# Role of blood pressure in development of early retinopathy in adolescents with type 1 diabetes: prospective cohort study

**DOI:** 10.1136/bmj.a918

**Published:** 2008-08-26

**Authors:** Patricia Herold Gallego, Maria E Craig, Stephen Hing, Kim C Donaghue

**Affiliations:** 1 Institute of Endocrinology and Diabetes, Children’s Hospital at Westmead, Locked Bag 4001, Westmead, NSW 2145, Australia; 2 Discipline of Paediatrics and Child Health, University of Sydney, NSW 2006, Australia; 3 School of Women’s and Children’s Health, University of New South Wales, Sydney NSW 2052

## Abstract

**Objective** To examine the relation between blood pressure and the development of early retinopathy in adolescents with childhood onset type 1 diabetes.

**Design** Prospective cohort study.

**Setting** Diabetes Complications Assessment Service at the Children’s Hospital at Westmead, Sydney, Australia.

**Participants** 1869 patients with type 1 diabetes (54% female) screened for retinopathy with baseline median age 13.4 (interquartile range 12.0-15.2) years, duration 4.9 (3.1-7.0) years, and albumin excretion rate of 4.4 (3.1-6.8) μg/min plus a subgroup of 1093 patients retinopathy-free at baseline and followed for a median 4.1 (2.4-6.6) years.

**Main outcome measures** Early background retinopathy; blood pressure.

**Results** Overall, retinopathy developed in 673 (36%) participants at any time point. In the retinopathy-free group, higher systolic blood pressure (odds ratio 1.01, 95% confidence interval 1.003 to 1.02) and diastolic blood pressure (1.01, 1.002 to 1.03) were predictors of retinopathy, after adjustment for albumin excretion rate (1.27, 1.13 to 1.42), haemoglobin A_1c_ (1.08, 1.02 to 1.15), duration of diabetes (1.16, 1.13 to 1.19), age (1.13, 1.08 to 1.17), and height (0.98, 0.97 to 0.99). In a subgroup of 1025 patients with albumin excretion rate below 7.5 μg/min, the cumulative risk of retinopathy at 10 years’ duration of diabetes was higher for those with systolic blood pressure on or above the 90th centile compared with those below the 90th centile (58% *v* 35%, P=0.03). The risk was also higher for patients with diastolic blood pressure on or above the 90th centile compared with those below the 90th centile (57% *v* 35%, P=0.005).

**Conclusions** Both systolic and diastolic blood pressure are predictors of retinopathy and increase the probability of early retinopathy independently of incipient nephropathy in young patients with type 1 diabetes.

## Introduction

Diabetic retinopathy affects most patients with type 1 diabetes after 15 years’ duration,^1^ ^2^ although more recent data indicate a reduction in incidence of retinopathy.^3^ ^4^ ^5^ Retinopathy is common in young patients with diabetes: in a population based study from Australia, 24% of children and adolescents with type 1 diabetes had early background retinopathy after only six years’ duration of diabetes,^6^ and retinopathy was present in 27% of Swedish patients after 13 years’ duration.^7^

In addition to hyperglycaemia, hypertension has emerged as a risk factor for diabetic retinopathy and its progression in adults.^8^ ^9^ ^10^ Hyperglycaemia may assert its main effect by impairing autoregulation of retinal perfusion, thereby leaving the eye unprotected from systemic hypertension.^11^ Indeed, retinal blood flow is higher in patients with diabetic retinopathy than in patients without retinopathy and controls without diabetes.^12^ Furthermore, retinal arteriolar dilatation may be the first sign of retinopathy in young patients with type 1 diabetes.^13^

Retinopathy and nephropathy often coexist in patients with diabetes, so elevation of blood pressure may be due to nephropathy rather than essential hypertension. Normotensive patients with type 1 diabetes and nephropathy have worse retinal changes than hypertensive patients with normoalbuminuria,^14^ but whether blood pressure acts independently or in association with nephropathy in the development of retinopathy is unclear. Hypertension is an independent predictor of diabetic retinopathy in all clinical studies.^14^ ^15^ ^16^ ^17^ Moreover, great variability exists in the evidence supporting the role of systolic or diastolic blood pressure in the development of diabetic retinopathy.^18^ ^19^ ^20^ ^21^ ^22^ Whether the relation is continuous or related only to a threshold effect of high blood pressure is also unclear.

Adolescents with diabetes are an ideal group in which to study the effect of blood pressure on the very early development of retinopathy, because of the absence of coexistent disease, smoking, and treatment with other drugs in most patients. We hypothesised that blood pressure as a continuous variable is a predictor of early development of retinopathy and that this relation is present even in the absence of incipient nephropathy. Hence, we examined the association between systolic and diastolic blood pressure and the development of early retinopathy in a large cohort of adolescents with type 1 diabetes.

## Methods

This study included 1869 adolescents aged under 15 (54% female) with a clinical diagnosis of type 1 diabetes who had retinal screening at the Diabetes Complications Assessment Service at the Children’s Hospital at Westmead (Sydney, New South Wales, Australia) between 1989 and 2007. We excluded patients with secondary causes of diabetes (pancreatectomy, cystic fibrosis, type 2 diabetes mellitus). Participants in this study represent 45% of patients enrolled on the NSW type 1 diabetes register^23^; they did not differ from non-participants by age, sex, or urban/rural location. All participants gave writteninformed consent.

### Screening for complications of diabetes

We screened adolescents for complications of diabetes according to established guidelines, which recommended annual assessment beginning five years after diagnosis (or age 11, whichever is earlier) in patients with prepubertal onset of diabetes and two years after diagnosis in patients with pubertal onset.^24^

We assessed retinopathy by fundal photography with a Topcon Fundus camera (TRC 50-VT, Tokyo Optical Co, Tokyo) after dilatation of the pupils with cyclopentolate 1% and phenylephrine 2.5%. We took non-simultaneous photographic pairs of seven standardised fields in each eye and then viewed them with a Donaldson Stereoviewer, which provided a three dimensional representation of the fundus and enabled microaneurysms to be more easily distinguished from haemorrhages and artefacts. From September 2004 we used the IMAGEnet 2000 Lite system to digitalise images. One experienced paediatric ophthalmologist (SH) graded the photographs according to the early treatment diabetic retinopathy study (ETDRS) adaptation of the modified Airlie House classification of diabetic retinopathy.^25^ We defined retinopathy as the presence of at least one microaneurysm or one haemorrhage (grade 21). For use as normal controls, we photographed 36 adolescents without diabetes; the grader was blinded to their non-diabetic status. These controls had no signs of microaneurysm or haemorrhage. We measured regrading agreement for 40 randomly selected participants (80 individual eyes) on two different occasions (1990-4 and 2001); weighted κ values of 0.76 and 0.88 represented excellent agreement.^26^

We determined albumin excretion rate from three consecutive timed overnight urine specimens. We used polyclonal radioimmunoassay (Pharmacia RIA, Beckman Coulter, Australia) to measure urinary albumin from 1989 to March 2000. From April 2000, the laboratory changed to nephelometric assay using an IMMAGE analyser (IMMAGE=(0.8734×radioimmunoassay value)−0.501; *r*=0.99). We defined microalbuminuria as albumin excretion rate ≥20 and <200 μg/min in two out of three samples. We defined normoalbuminuria as mean albumin excretion rate <7.5 μg/min in all urine samples.

At each complications assessment, we assessed glycaemic control by measuring glycated haemoglobin colorimetrically^27^ before February 1994 and subsequently by haemoglobin A_1c_ with high performance liquid chromatography (Diamat Bio-Rad analyser, Bio-Rad, Herculus, CA; non-diabetic range 4-6%). We converted glycated haemoglobin values to haemoglobin A_1c_ (Diamat=1.9088+0.0043×GHb; *r*=0.92). The interassay coefficients of variation were 1.1% and 1.2% for values of haemoglobin A_1c_ of 5.95% and 9.76%.

### Blood pressure and other measurements

We used auscultation with a conventional mercury sphygmomanometer device and appropriate cuff sizes to measure systolic and diastolic blood pressure twice in the seated position after five minutes’ rest. We used the right arm for all measurements. We took the onset of the first and fifth Korotkoff phases as systolic and diastolic blood pressure. We report blood pressure measurements in mm Hg or in Z scores after adjustments for age and sex, as recommended in childhood and adolescence.^28^ Anthropometric data included height (cm), weight (kg), and body mass index (kg/m^2^). We determined Z scores for body mass index by using age and sex related reference standards.^29^

### Statistical analysis

We present descriptive statistics as mean and standard deviation for normally distributed data or median and interquartile range when distributions were skewed. We used the χ^2^ test to compare groups for categorical variables. We evaluated differences between independent samples by using Student’s *t* test if variables were normally distributed or Mann-Whitney U test for skewed data.

#### Cox proportional hazard regression and Kaplan-Meier survival curves

We used Cox proportional hazards regression to examine predictors of the first retinopathy event in all participants (n=1869), with duration of diabetes as the time variable. Covariates included were systolic and diastolic blood pressure (mm Hg), sex, age, height (cm), haemoglobin A_1c,_ and BMI Z score or BMI >95th centile (used as definition for obesity in adolescents).^30^ We examined clinically relevant interaction terms (blood pressure*age, blood pressure*sex, blood pressure*age*sex, and age*sex), but they were not significant. We included quadratic terms for systolic and diastolic blood pressure to test for curvature. We report only significant results, expressed as hazard ratio and 95% confidence interval.

To exclude the effect of early elevation of albumin excretion on the development of retinopathy, we used Kaplan-Meier survival curves to estimate the probability of developing the first retinopathy event in participants who had albumin excretion rate <7.5 μg/min (n=1025). We used high-normal cut off for systolic and diastolic blood pressure, defined as blood pressure on or above the 90th centile,^28^ as factors and used the log-rank test to compare survival times.

#### Generalised estimating equations

We did longitudinal analysis in participants who were retinopathy-free at baseline and followed prospectively by using generalised estimating equations so that correlations between repeated measures for a given patient could be taken into account.^31^ In this model, we examined different covariates as predictors of the outcome variable, presence or absence of retinopathy at any time. We used systolic and diastolic blood pressures (mm Hg), sex, age, duration of diabetes, haemoglobin A_1c_, albumin excretion rate (expressed as log albumin excretion rate), height (cm), and Z score for body mass index as covariates. We examined clinically relevant interaction terms (as above), but they were not significant. To test for curvature, a quadratic blood pressure term was included in the analysis. We report significant results as odds ratio and 95% confidence interval.

## Results

### Clinical characteristics

For the whole group (n=1869, 54% female), median age was 13.4 years (interquartile range 12.0-15.2), duration of diabetes was 4.9 (3.1-7.0) years, albumin excretion rate was 4.4 (3.1-6.8) μg/min, and haemoglobin A_1c_ was 8.4% (7.7-9.3%) at first assessment. Overall, 673 (36%) participants developed retinopathy at any time during the follow-up. Moderate to severe non-proliferative retinopathy developed in 35 participants, and only one patient developed proliferative retinopathy during the study.

Table 1 shows baseline clinical characteristics for the total cohort stratified by retinal status. We compared participants who never developed retinopathy with those who developed retinopathy at any stage during the follow-up and found no difference in baseline age, albumin excretion rate, or prevalence of microalbuminuria. However, systolic blood pressure, diastolic blood pressure, duration of diabetes, and haemoglobin A_1c_ were higher in patients who developed retinopathy.

**Table 1 tbl1:** Baseline clinical characteristics of patients with type 1 diabetes mellitus in relation to development of retinopathy during follow-up. Values are number (percentage) or median (interquartile range) unless stated otherwise

Characteristic	No retinopathy (n=1196)	Retinopathy (n=673)	P value
Female	618 (51.7)	384 (57.1)	0.03
Mean (SD) age (years)	13.5 (2.34)	13.4 (2.42)	0.35
Duration of diabetes (years)	4.3 (2.9-6.2)	5.7 (3.4-8.3)	<0.001
Haemoglobin A_1c_ (%)	8.3 (7.6-9.2)	8.6 (7.8-9.5)	<0.001
Albumin excretion rate (μg/min)	4.3 (3.0-6.8)	4.4 (3.3-6.8)	0.18
Systolic blood pressure (mm Hg)	110 (100-120)	110 (105-120)	0.001
Systolic blood pressure Z score	0.19 (−0.64-0.82)	0.23 (−0.25-0.91)	<0.001
Diastolic blood pressure (mm Hg)	65 (60-70)	70 (60-70)	<0.001
Diastolic blood pressure Z score	0.26 (−0.35-0.81)	0.43 (−0.21-0.91)	<0.001
Body mass index (kg/m^2^)	21 (18.8-23.8))	20.3 (18.6-22.9)	<0.001
Body mass index (Z score)	0.67 (0.11-1.20)	0.48 (−0.01-1.01)	<0.001
Microalbuminuria	22/1055 (2.1)	13/542 (2.4)	0.72

In all, 1231 (66%) participants had more than one visit, with median follow-up of 4.1 (3.1-7.2 ) years and a median number of visits of 3 (2-5). The incidence of retinopathy was 14.5 per 100 person years of follow-up. In this group, 138 participants had retinopathy at baseline, of whom 50 (36%) regressed, 56 (41%) were unchanged, and 32 (23%) progressed during follow-up.

#### Cox proportional hazard regression

In Cox proportional hazard regression (n=1869), systolic blood pressure, female sex, haemoglobin A_1c_, age, and body mass index above 95th centile were significantly associated with a higher cumulative risk of retinopathy (table 2). The quadratic term for blood pressure was not significant, suggesting a linear effect.

**Table 2 tbl2:** Results of Cox proportional hazards regression for development of first event of retinopathy, with duration of diabetes as time variable, in young patients with type 1 diabetes mellitus (n=1869)

Covariates	Hazard ratio (95% CI)	P value
Systolic blood pressure (mm Hg)	1.02 (1.01 to 1.03)	<0.001
Female sex	1.29 (1.10 to 1.51)	0.002
Age (years)	0.83 (0.81 to 0.85)	<0.001
Haemoglobin A_1c_	1.06 (1.01 to 1.12)	0.02
Body mass index >95th centile	1.34 (1.01 to 1.78)	0.04

Among participants with albumin excretion rate <7.5 μg/min (n=1025), the cumulative risk of retinopathy at 10 years’ duration of diabetes was higher for those with systolic blood pressure on or above the 90th centile than for those below the 90th centile (58% *v* 35%, P=0.03) (fig 1). Similarly, diastolic blood pressure on or above the 90th centile conferred a higher risk compared with those below the 90th centile (57% *v* 35%, p=0.005) (fig 2).

**Figure fig1:**
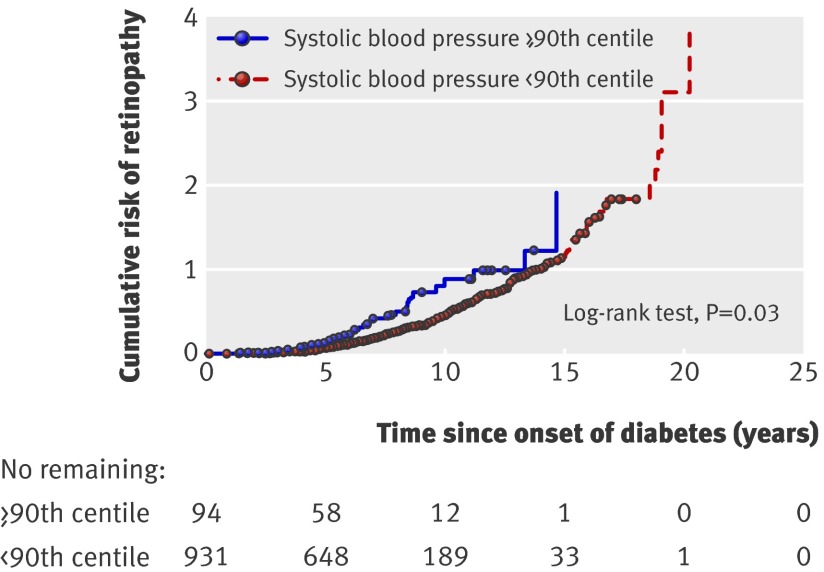
**Fig 1** Cumulative probability of first event of retinopathy for each year since onset of diabetes in patients with albumin excretion rate always below 7.5 μg/min (n=1025). Patients were grouped according to systolic blood pressure below or on/above 90th centile

**Figure fig2:**
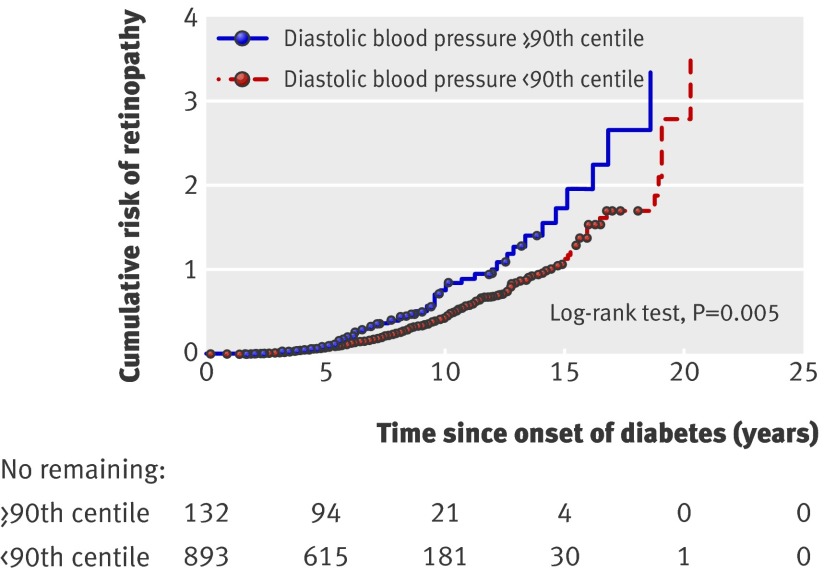
**Fig 2** Cumulative probability of first event of retinopathy for each year since onset of diabetes in patients with albumin excretion rate always below 7.5 μg/min (n=1025). Patients were grouped according to diastolic blood pressure below or on/above 90th centile

### Longitudinal analysis

By using generalised estimating equations, we analysed two subgroups of participants. The first group included all participants with more than one visit who were retinopathy-free at baseline (n=1093). Retinopathy was significantly associated with higher systolic and diastolic blood pressure. Age, duration of diabetes, haemoglobin A_1c_, height, and log albumin excretion rate were also significant predictors of retinopathy (model 1, table 3).

**Table 3 tbl3:** Factors associated with occurrence of retinopathy in adolescents with type 1 diabetes mellitus who were retinopathy-free at baseline: longitudinal analysis using generalised estimating equations (n=1093)

Covariates	Odds ratio (95% CI)	P value
Model 1*		
Systolic blood pressure (mm Hg)	1.01 (1.003 to 1.02)	0.008
Diastolic blood pressure (mm Hg)	1.01 (1.002 to 1.03)	0.02
Age (years)	1.13 (1.08 to 1.17)	<0.001
Duration (years)	1.16 (1.13 to 1.19)	<0.001
Haemoglobin A_1c_	1.08 ( 1.02 to 1.15)	0.01
Log albumin excretion rate	1.27 (1.13 to 1.42)	<0.001
Height (cm)	0.98 ( 0.97 to 0.99)	<0.001
**Model 2†**		
Diastolic blood pressure (mm Hg)	1.02 (1.01 to 1.04)	0.004
Age (years)	1.12 (1.06 to 1.18)	<0.001
Duration (years)	1.14 (1.09 to 1.18)	<0.001
Haemoglobin A_1c_	1.14 (1.03 to 1.26)	0.009

*Included systolic and diastolic blood pressure measurements from patients who were retinopathy-free at baseline (n=1093) along with other covariates.

†Included systolic and diastolic blood pressure measurements from patients who were retinopathy-free at baseline and had albumin excretion rate consistently below 7.5 μg/min throughout follow-up (n=594).

The second group consisted of 594 participants with more than one visit who were retinopathy-free at baseline and had albumin excretion rate consistently <7.5 μg/min. Results showed that diastolic blood pressure, in addition to age, duration of diabetes, and haemoglobin A_1c_, predicted retinopathy (model 2, table 3). In both groups, quadratic terms for systolic and diastolic blood pressure were not significant, suggesting a linear rather than a threshold effect of blood pressure as a predictor for retinopathy.

## Discussion

This longitudinal cohort study showed that higher blood pressure contributes to the early development of retinopathy in adolescents with type 1 diabetes, independent of other known risk factors. Both systolic and diastolic blood pressure contributed to risk of retinopathy. Not only was the effect independent of glycaemic control but it was independent of early elevation of albumin excretion. This supports the hypothesis that blood pressure acts independently, rather than by association with nephropathy, in the development of retinopathy. In addition, these results suggest that the effect of blood pressure as a predictor for retinopathy is linear rather than a threshold effect. In the longitudinal cohort, a 10 mm Hg higher systolic blood pressure was associated with a 3-20% higher risk of retinopathy; a 10 mm Hg higher diastolic blood pressure increased the risk for retinopathy by 2-30% after adjustments for age, sex, and albumin excretion rate. An increase of 1% in haemoglobin A_1c_ was associated with a 2-15% higher risk of retinopathy, and an extra one year’s duration of diabetes was associated with a 13-19% greater risk of retinopathy.

### Prevalence of retinopathy

Overall, we reported that 36% of these patients developed retinopathy. Some investigators have reported a lower prevalence of retinopathy in the young population than we saw in our study. Divergent results may be explained by differences in age, sample size, study design, and methods of screening for retinopathy. Kernell and colleagues reported a prevalence of retinopathy of 14.5% in adolescents studied cross sectionally, but retinopathy was assessed by the less sensitive three field photography instead of seven fundal photography, the gold standard tool for retinopathy screening.^32^ Nordwall and colleagues reported a prevalence of retinopathy of 27% in 80 children and adolescents with type 1 diabetes mellitus aged 7-22 years despite intensive management over a follow-up of 13 years.^7^

### Systolic and diastolic blood pressure and retinopathy

The strength of our study is the large cohort of young patients followed for a median of four years, enabling exploratory analyses of risk factors over time. The sample consisted of adolescents who were not on any other drugs except insulin, thereby reducing the effect of comorbidities on risk of retinopathy. Our findings extend the reports of the Wisconsin epidemiologic study of diabetic retinopathy, in which higher baseline systolic blood pressure was associated with increased risk of retinopathy 10 years later in the young onset diabetes group (<30 years) and higher diastolic blood pressure was associated with progression of retinopathy at 14 years.^9^ ^33^ Macroproteinuria at baseline was not significant in their models, but this is a late measure of renal disease.

A potential weakness of the study is the use of auscultation without a random zero machine. However, the physicians taking the blood pressure were not aware of the retinal status of the patients.

### Blood pressure as a predictor of retinopathy in the absence of incipient nephropathy

Our study has shown that higher diastolic blood pressure was still an independent predictor of retinopathy in adolescents with albumin excretion rate consistently below 7.5 μg/min, an earlier marker of incipient renal disease. Conversely, albumin excretion rate also predicted development of retinopathy in the entire cohort. Although antihypertensive agents prevent the progression from normoalbuminuria to microalbuminuria in patients with and without hypertension,^34^ whether treatment of normotensive young patients with type 1 diabetes and normoalbuminuria will influence retinopathy risk is not known.

Hypertension has been implicated in the development of diabetic retinopathy, on the basis of data from observational studies, which have not always controlled for other contributing factors. Interventional studies with angiotensin converting enzyme inhibitors have shown a reduction in progression of retinopathy in adults with type 1 diabetes,^35^ in the absence of hypertension, and in type 2 diabetes.^36^ ^37^ Although this suggests that lowering of high blood pressure influences the risk of retinopathy, these drugs may have benefits on the eye independent of their antihypertensive properties, possibly by affecting local production of angiotensin converting enzyme by retinal vascular endothelial cells.^38^

The definition of hypertension is arbitrary. In adults with diabetes, lower targets for intervention have been recommended than in the general population,^39^ and intensive blood pressure control in normotensive patients with diabetes may slow the progression of nephropathy and retinopathy.^34^ ^40^ However, the ADVANCE study found no significant reduction in retinopathy at lower levels of blood pressure elevation.^31^

### Conclusions

This study shows that a continuous relation exists between blood pressure and risk of retinopathy. The clinical care of adolescents with type 1 diabetes is often shared between specialists and general practitioners. These findings highlight the importance of close blood pressure monitoring by all professionals involved in the management of adolescents with diabetes, regardless of urinary albumin excretion and glycaemic control, and indicate that lowering blood pressure, even in patients without hypertension or microalbuminuria, may improve retinal outcomes in diabetes. However, evidence for this proposal is lacking and interventional trials are needed to test the risks and benefits of lowering blood pressure in children and adolescents with type 1 diabetes.

**What is already known on this topic**

No longitudinal data exist on the effect of blood pressure on the development of retinopathy in children and adolescents with type 1 diabetes
In adults with diabetes, hypertension has emerged as a risk factor for diabetic retinopathy and its progression

**What this study adds**

Higher systolic and diastolic blood pressure contribute to the early development of diabetic retinopathy independent of glycaemic control, duration of diabetes, and albumin excretion
Blood pressure had a continuous effect rather than a threshold effect on risk of retinopathy, suggesting that lower blood pressure protects the eye in diabetes
This relation has potential implications for blood pressure lowering treatment for the prevention of diabetic retinopathy in adolescents with type 1 diabetes

## Notes

Contributors: PHG was involved in statistical analysis, data interpretation, and drafting of the manuscript. MEC was involved in data collection, statistical analysis, data interpretation, and revision of the manuscript. SH was involved in data collection, including retinal photography assessment. KCD was involved in the conception and design of the study, collection and interpretation of data, and writing and revision of the manuscript. KCD is the guarantor.

Ethical approval: Ethics committee of Children’s Hospital at Westmead, Sydney.

Provenance and peer review: Not commissioned; externally peer reviewed.

## Notes

**Cite this as:** *BMJ* 2008;337:a918
